# Gut–Joint Axis: The Role of Exercise on Gut Microbiota and Acetic Acid Modulation in Obesity-Associated Osteoarthritis Rats

**DOI:** 10.3390/metabo16070452

**Published:** 2026-06-27

**Authors:** Ruimin Chi, Xiaoxia Hao, Jiamin Lin, Jiawei Liu, Bingjin Liu, Tao Xu

**Affiliations:** 1 School of Medicine and Pharmaceutical Engineering, Taizhou Vocational and Technical College, Taizhou 318000, China; 2 Department of Rehabilitation, Tongji Hospital, Tongji Medical College, Huazhong University of Science and Technology, Wuhan 430030, China; 3 Department of Plastic Surgery, Taizhou Hospital of Zhejiang Province, Wenzhou Medical University, Taizhou 318000, China

**Keywords:** obesity-associated osteoarthritis, exercise, gut microbiota, acetic acid

## Abstract

**Objective:** This study aims to explore the effects of exercise on gut microbiota and short-chain fatty acid (SCFA) metabolism, as well as its relationships with joint degeneration. **Methods:** To explore the impact of exercise on obesity-associated osteoarthritis (OA), rats were fed a high-fat/high-sucrose (HFS) diet with or without exercise. Histological staining and micro-computed tomography (micro-CT) were used to assess the effects of exercise on articular cartilage and subchondral bone. 16S rRNA sequencing and Gas Chromatography–Mass Spectrometry (GC-MS) were performed to analyze alteration in fecal gut microbiota and serum SCFAs. **Results:** Exercise prevented articular cartilage degeneration and subchondral bone loss in the HFS diet with exercise (HE) group compared to the HFS-diet control sedentary (HS) group. Gut microbiota analysis revealed that exercise reduced the relative abundances of families *Lachnospiraceae*, *Ruminococcaceae*, genera *Ruminococcus*, *Colidextribacter*, *Caproiciproducens*, and the unidentified genus of *Lachnospiraceae*, while increased the relative abundance of genus *Akkermansia*. Metabolomic analysis indicated exercise prevented AA reduction in the HE group. In addition, the level of AA was negatively correlated with OA severity and with the abundances of families *Lachnospiraceae* and *Ruminococcaceae*, genus *Colidextribacter* and the unidentified genus of *Lachnospiraceae*. **Conclusions:** Exercise effectively preserves the integrity of the cartilage–subchondral bone unit. The observed modifications in gut microbiota and AA levels following exercise intervention may be associated with the protective mechanisms against obesity-associated OA.

## 1. Introduction

Osteoarthritis (OA) is a commonly occurring musculoskeletal disease characterized by the chronic degenerative changes involving whole joints, leading to pain and even affecting quality of life [1]. The pathogenesis of OA is multifactorial, involving various risk factors and manifesting in multiple subtypes [2]. Among these factors, obesity stands out as one of the most significant and modifiable risk factors, making it a focal point in OA research [3,4]. Obesity-associated OA is considered to be a phenotype of metabolic syndrome-associated OA (MetS-OA), which arises as a complication of underlying metabolic disorders [5]. Obesity not only increases the load on weight-bearing joints but also leads to joint malalignment and reduced periarticular muscle strength, thereby augmenting mechanical stress on joints, compromising joint load-bearing capacity, and ultimately causing articular cartilage degeneration and osteoarthritis (OA) [6,7,8,9,10,11]. However, it fails to explain the elevated incidence of OA in non-weight-bearing joints such as in the hands [12]. Growing evidence suggests that systemic inflammation resulting from obesity is an important pathophysiological mechanism bridging obesity and OA, regulating lipid metabolism, cartilage degradation and bone loss [3,4].

The gut microbiota, the totality of microbes in the gastrointestinal tract, plays a pivotal role in regulating host endocrine and immune systems [9,10,11]. A comprehensive review encompassing 10 human studies and 21 animal studies has reached a consistent conclusion that gut microbiota dysbiosis could aggravate OA [13]. Numerous studies indicate that alterations in gut microbiota composition, alongside increased intestinal permeability and low-grade inflammation, may collectively contribute to the progression of degenerative joint processes [13,14,15,16]. The mechanisms through which the microbiota influences immune regulation and host metabolism are increasingly recognized as multifactorial, encompassing converging pathways involving SCFAs, tryptophan metabolites, and bile acids [17]. In a destabilization of the medial meniscus (DMM) model, germ-free (GF) mice displayed attenuated joint pathology relative to specific pathogen-free (SPF) counterparts, implicating the role of the gut microbiota in the etiopathogenesis of OA [18]. Antibiotic-mediated perturbation of the gut microbiota has been shown to attenuate DMM-induced OA progression in mice, concomitant with reductions in circulating lipopolysaccharide (LPS) and suppression of systemic inflammatory cascades [19]. In addition, Streptococcus thermophilus (TCI633), a hyaluronic acid-producing bacterium, has been shown to improve serum collagen type II C-telopeptide and C-reactive protein levels, alleviating OA progression in patients [20]. A cross-sectional analysis of two independent cohorts revealed that symptomatic hand osteoarthritis (SHOA) was associated with altered microbial tryptophan biosynthesis and corresponding shifts in tryptophan metabolites [21]. Collectively, these data underscore a robust interplay among gut microbiota, microbiota-derived metabolites, and OA etiopathogenesis. SCFAs are collections of saturated fatty acids with 1~6 carbon atoms that are created by microbial organisms, and are involved in various biological functions [22,23]. SCFAs are able to maintain intestinal barrier integrity, influence gastrointestinal motility, and modulate adaptive immune responses through direct or indirect regulation of T cell differentiation and proliferation [22]. In addition, SCFAs also exert regulatory effects on energy metabolism [23]. In GF mice, supplementation with fermentable fibers can improve microbiota dysbiosis caused by a high-fat diet, promote the production of SCFAs and Interleukin-22 (IL-22), thereby preventing metabolic syndrome [24]. Therefore, manipulating the gut microbiota to influence SCFA metabolism may present a novel strategy for managing OA, which can be achieved by adjusting diet, lifestyle and exercise [25].

Exercise is one of the important therapeutic strategies for OA. It can alleviate the onset and progression of OA by improving the morphology and structure of articular cartilage and subchondral bone, enhancing muscle strength, improving joint stability, and reducing systemic inflammation [26,27]. Emerging evidence indicates that gut microbiota alteration represents an additional pathway through which exercise may influence host health [25,28]. The salutary effects of exercise are mediated by diverse mechanisms, including remodeling bile acid profiles, augmenting SCFA synthesis, attenuating TLR signaling, strengthening mucosal immunity through IgA production, hastening gastrointestinal transit, and engaging the HPA axis [29]. These microbiota-dependent pathways underlie the therapeutic benefits of exercise in various disease states, including obesity, diabetes, and environmental toxicant-induced toxicity [29,30,31,32]. Notably, the alteration in the gut microbiota and its metabolites’ SCFAs occurred after aerobic exercise for 6 weeks [33]. Munukka et al. reported that six weeks of exercise induced marked taxonomic restructuring in the gut microbiota of previously sedentary overweight women, characterized by a relative expansion of genus *Akkermansia* and a concomitant contraction of phylum *Proteobacteria* [34]. In the specific context of OA, our previous study has demonstrated that moderate treadmill exercise (15 m/min, 30 min/day, 5 days/week for 8 weeks) exerts a protective effect against OA progression by specifically reducing the abundance of pro-inflammatory phylum *Fusobacteria* and decreasing the levels of their metabolite, lipopolysaccharide (LPS) [15]. This exercise protocol remodels the OA-related gut microbiome, reduces microbiota-derived LPS, and subsequently downregulates systemic pro-inflammatory factors, ultimately alleviating systemic inflammation and improving joint degeneration [15,35]. A recent study has further expanded this understanding, showing that a combined intervention of progressive moderate treadmill exercise (30 min/day, 5 days/week for 12 weeks) and prebiotic fiber can more effectively maintain cartilage integrity and prevent high-fat-diet-induced joint injury by modulating the gut microbiota [36]. However, it remains elusive whether the microbiota-derived SCFAs are associated with the mechanism of exercise influencing the joint during obesity-associated OA development.

Based on the aforementioned evidence, we hypothesize that moderate treadmill exercise may attenuate HFS-diet-induced joint degeneration, with this protective effect potentially being associated with gut microbiota modulation and enhanced circulating SCFA availability. To test this hypothesis, we investigated the effects of treadmill walking (15 m/min, 30 min/day, 5 days/week for 12 weeks) on the knee joint, gut microbiota profiles, and serum SCFA levels in obesity-associated OA rats.

## 2. Methods

### 2.1. Animals

A total of 24 male Sprague-Dawley rats (8 weeks old) were purchased from the Weitong Lihua Animal Co., Ltd. (Beijing, China), with a qualified number of 422023600004695. The rats were housed in ventilated cages individually and bred in the specific pathogen-free (SPF) condition (12 h light/dark cycle, a consistent temperature of 21–23 °C). All rats were allowed to move and eat freely in the cages. All protocols in this animal experiment have been approved by the Experimental Animal Ethics Committee of Tongji Medical College, Huazhong University of Science and Technology (approval code: TJH-202305009; date of approval: 9 May 2023).

### 2.2. Animal Design

**Figure 1:**
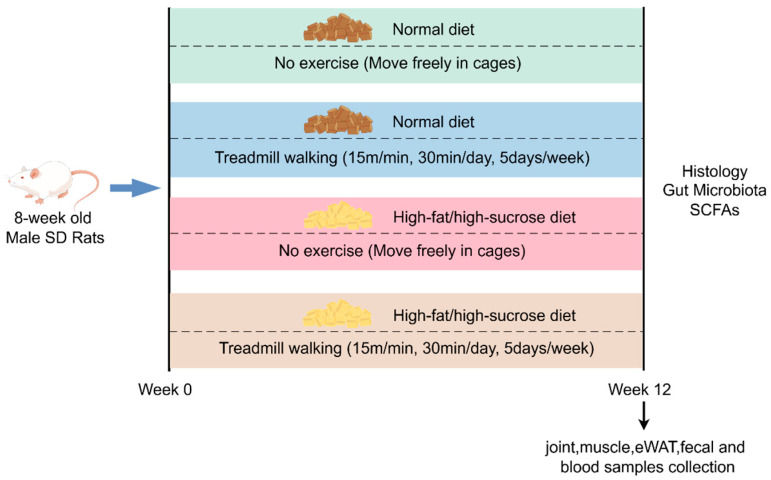
Animal experimental protocols. 24 rats were randomly divided into 4 groups: normal diet control sedentary group (NS), normal diet with exercise group (NE), high-fat/high-sucrose-diet control sedentary group (HS) and high-fat/high-sucrose diet with exercise group (HE). The exercise regimen of this study was to exercise at a speed of 15 m/min on the treadmill for 30 min/day, 5 days/week. Prior to the formal intervention, rats underwent a 1-week adaptation period (10 m/min, 10 min/day, 5 days/week) to acclimate to the treadmill apparatus. After 12 weeks of intervention, the rats were euthanized and knee joint, muscle, eWAT, fresh fecal and blood samples were collected for analysis.

The procedure of this experiment is shown in Figure 1. The 24 rats were randomly divided into four groups: normal diet control sedentary group (NS), normal diet with exercise group (NE), high-fat/high-sucrose-diet control sedentary group (HS) and high-fat/high-sucrose diet with exercise group (HE). With a preset α of 0.05 and 80% power, five animals per group represented the minimum sample size necessary to identify a meaningful minimal difference in knee joint histological scores. This estimate was generated via G*Power Software (version 3.0.10, Germany) [37], with reference to data reported in a prior study [38]. The normal diet refers to a diet that consists of 5% fat and 47.5% carbohydrates (sucrose is only 4%) of total weight. The HFS diet refers to a diet containing 20% fat, 50% sucrose, 20% protein, and 10% fiber and micronutrients by weight (custom Diet #102412, Dyets, Wuxi, China). The exercise regimen was set to a speed of 15 m/min for 30 min/day, 5 days/week. Prior to the formal intervention, rats underwent a 1-week adaptation period (10 m/min, 10 min/day, 5 days/week) to acclimate to the treadmill apparatus. After 12 weeks of intervention, the rats were euthanized using high-concentration carbon dioxide. Knee joint, quadricep muscle, gastrocnemius muscle, and epididymal white adipose tissue (eWAT) were collected for analysis. Prior to execution, we harvested fresh fecal samples from each rat immediately after excretion and then stored them at −80 °C. In addition, blood samples are taken by heart puncture.

### 2.3. Body Weight and Fasting Blood Glucose

The rats were weighed once a week. The fasting blood glucose was measured every two weeks. Rats were fasted for 14~16 h prior to the measurement. After the fasting period, fasting blood glucose was measured using a Roche glucometer via tail vein blood sampling.

### 2.4. Micro-CT Analysis of Subchondral Bone

A micro-CT (micro-CT 50 Scanco Medical, Bassersdorf, Switzerland) system was used to scan the knee joints of all rats. The parameters were as follows: voltage of 100 kV, resolution of 10.5 µm, and current of 98 µA. After the scan was completed, the three-dimensional (3D) images were reconstructed and the data were calculated to analyze the change in subchondral bone according to the method previously described in a study by the same authors [15,16]. The data were conducted by three independent, experienced investigators in a masked manner, with the mean value calculated for each sample.

### 2.5. Histological Analysis of Joints

After micro-CT analysis, the knee joints were decalcified for 1 month in 10% ethylenediaminetetraacetic acid (EDTA) solution and embedded in the sagittal plane using paraffin. Finally, 4 μm thick sections were obtained for histological analysis. Safranin O-Fast Green staining of tissue sections was used to measure the variation in cartilage. The Osteoarthritis Research Society International (OARSI) scoring system (0–6) [39] and modified Mankin scoring system (0–14) [40] were employed to grade cartilage degeneration. These criteria involve evaluation of chondrocyte loss, articular cartilage structural changes, and matrix fibrillation or loss. Scoring was conducted by three independent, experienced investigators in a masked manner, with the mean value calculated for each section. A single sample was represented by the average score of ten randomly selected sections.

### 2.6. Histological Analysis of Muscle and eWAT

The intact quadricep muscle, gastrocnemius muscle and eWAT were fixed with paraformaldehyde, dehydrated with ethanol, and embedded with paraffin. Then, those samples were sectioned and hematoxylin and eosin (HE) staining was performed according to the previously published study [27,41].

### 2.7. 16S rRNA Gene Sequencing Process

Total genomic DNA was isolated from the samples with the CTAB method, and its purity and concentration were measured on an agarose gel. Then, the sample was appropriately diluted to an approximate concentration based on rough estimation by agarose gel electrophoresis. The diluted genomic DNA was used as a template for PCR amplification with barcoded region-specific primers, Phusion^®^ High-Fidelity PCR Master Mix with GC Buffer (New England Biolabs, Ipswich, MA, USA) and a high-efficiency high-fidelity polymerase to ensure amplification efficiency and accuracy. The 341F and 806R primers (Supplementary Table S1) were used to amplify the V3–V4 region of the 16S rRNA genes. The PCR program was the following: initial denaturation at 98 °C for 1 min; 30 cycles including 98 °C for 10 s, 50 °C for 30 s, and 72 °C for 30 s; and final extension for 5 min at 72 C. The PCR products were then purified using the Gel Extraction Kit (Qiagen, Hilden, Germany). Library construction was performed using the TruSeq^®^ DNA PCR-Free Sample Preparation Kit (Illumina, San Diego, CA, USA). The constructed library was quantified by Qubit^®^ 2.0 Fluorometer (Thermo Fisher Scientific, Waltham, MA, USA) and qPCR. After passing quality control, the libraries were sequenced on the NovaSeq 6000 platform (Illumina, San Diego, CA, USA).

### 2.8. Analysis of Sequencing Data

Based on the barcode sequences and PCR amplification primer sequences, individual sample data were demultiplexed from the raw sequencing output, and barcode and primer sequences were trimmed. Raw reads were filtered using fastp (v0.22.0) to obtain high-quality reads with the following filtering criteria: automatic detection and removal of adapter sequences; removal of reads containing ≥15 ambiguous N bases; removal of reads with >50% low-quality bases (quality score ≤ 20); truncation of reads at the position where the average quality score within a 4-base sliding window dropped below 20; removal of poly-G tails at the 3’ end; and removal of reads shorter than 150 bp. High-quality paired-end reads were then assembled using FLASH (v1.2.11) to generate high-quality tag data (Clean Tags). Chimera sequences were detected by aligning tag sequences against the species annotation database using vsearch (v2.22.1), and subsequently removed to obtain the final effective data (Effective Tags). Using Deblur (v1.1.1) implemented in QIIME 2 (v2023.2), Hamming distances between sequences within each sample were compared against an upper-bound error curve, and amplicon sequence variants (ASVs) were obtained in combination with a greedy algorithm. Species annotation of ASV sequences was performed using the Mothur method (v1.48) against the SILVA 138.1 SSU rRNA database with a confidence threshold of 0.8–1. Taxonomic information was obtained, and community composition at each sample was summarized across all taxonomic levels: phylum, class, order, family, genus, and species. Finally, data from each sample were normalized by rarefaction to the minimum sequencing depth across samples. Subsequent alpha diversity and beta diversity analyses were based on these normalized data. A Venn diagram facilitated the comparison of ASVs across the groups. R software with the phyloseq (v1.40.0) and vegan (v2.6.2) packages was used to calculate the ACE, Chao1, Shannon and Simpson indices and the α diversity was measured using the Kruskal–Wallis test. The PCoA analysis was performed using the phyloseq package (v1.40.0) in R software. PERMANOVA was conducted using the adonis function in the vegan package (v2.6.2) in R (v4.2.0) with Bray–Curtis distance (method = “bray”) and 999 permutations (permutations = 999). Spearman correlation analysis was performed using the stats package in R (v4.2.0). OPLS-DA was performed using the MetaboAnalystR package in R (v4.2.0) with the OPLSR.Anal function. LEfSe analysis was performed using LEfSe software (v1.1.2) with a default LDA score threshold of 4.0 and *p* < 0.05 as the significance criteria. Differences in species composition between groups were analyzed using the Mann–Whitney U test.

### 2.9. SCFA Quantification

Serum samples were thawed and rotated for 1 min before analysis. Samples (50 μL) and phosphoric acid (0.5%, 100 μL) solution were added to the 1.5 mL centrifuge tube and vortexed at the speed of 2500 r/min for 3 min to mix. Then, the mixture was mixed with 200 μL Methyl tert-butyl ether (MTBE) by vortex at the speed of 2500 r/min for 3 min and ultrasound for 5 min. Finally, the mixture was centrifuged (12,000 r/min, 10 min, 4 °C) and the supernatant was collected for Gas Chromatography–Mass Spectrometry (GC-MS) analysis [42,43,44]. The SCFAs in serum were analyzed by GC-MS using Agilent 8890-7000D (Agilent Technologies, Santa Clara, CA, USA). The carrier gas was helium and the flow rate was 1.2 mL/min. The samples were injected in the split mode (5:1) and the injection volume was 1 μL. The temperature of oven was maintained at 50 °C for 1 min and increased to 220 °C at 18 °C/min for 5 min. The samples were measured in multi-reaction monitoring mode. The inlet temperature of the injector was 250 °C, and the temperature of the transfer line was 230 °C [44,45].

### 2.10. Statistical Analysis

Parametric data were analyzed using one-way ANOVA followed by Tukey’s post hoc test for multiple-group comparisons and Student’s *t*-test for two-group comparisons. Nonparametric data were analyzed using the Kruskal–Wallis H test for multiple-group comparisons and the Mann–Whitney U test for two-group comparisons. The data were analyzed using GraphPad Prism software (version 8.0, USA) and are shown as means and standard deviations. Correlations were conducted utilizing Spearman’s correlation in python (version 3.12.4). The difference was deemed statistically significant if the *p*-value < 0.05.

## 3. Results

### 3.1. Exercise Prevents Joint Degradation Induced by HFS Diet

**Figure 2:**
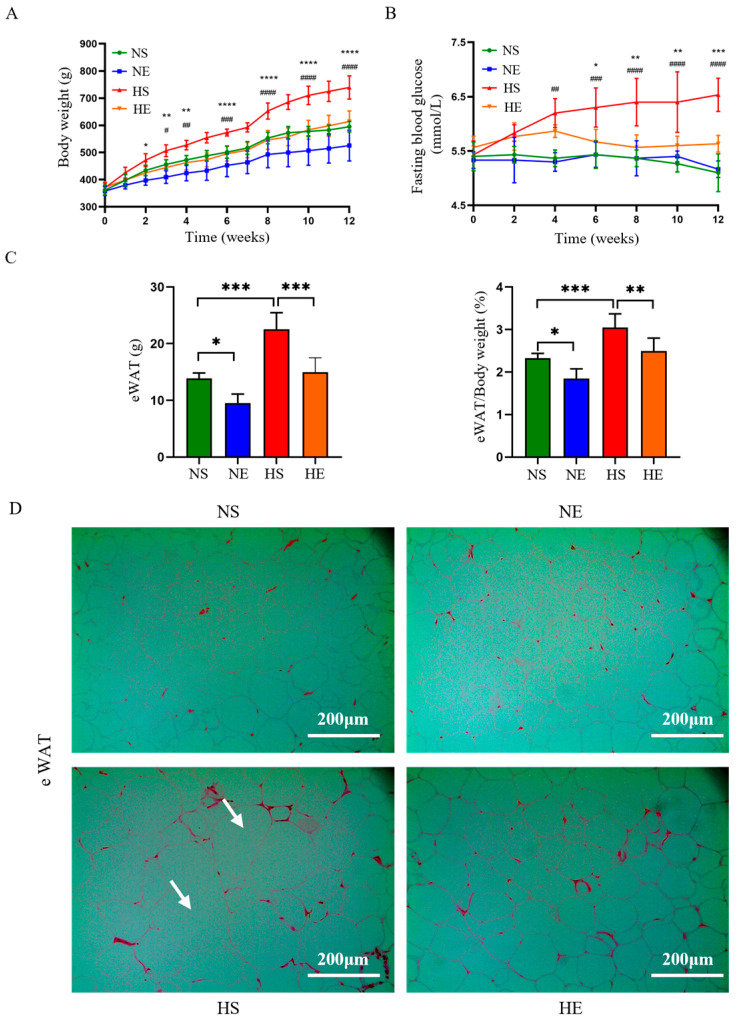
Exercise prevents obesity induced by HFS diet. (**A**,**B**) The body weight and fasting blood glucose of rats. # NS vs. HS, *p* < 0.05; ## NS vs. HS, *p* < 0.01; ### NS vs. HS, *p* < 0.001; #### NS vs. HS, *p* < 0.0001; * HS vs. HE, *p* < 0.05; ** HS vs. HE, *p* < 0.01; *** HS vs. HE, *p* < 0.001; **** HS vs. HE, *p* < 0.0001. (**C**) The eWAT weight and weight ratios of NS, NE, HS and HE groups. (**D**) HE staining of eWAT in each group (scale bar = 200 μm). White arrows indicate adipocytes with increased cell diameter. Data are presented as the mean ± SD, *n* = 6. * *p* < 0.05, ** *p* < 0.01, *** *p* < 0.001, **** *p* < 0.0001.

To verify the impact of exercise on obesity induced by the HFS diet, we recorded body weight and fasting blood glucose in rats. As shown in Figure 2A,B, the body weight and fasting blood glucose of the HS group were significantly higher than those of the NS group at the beginning of the third and fourth week. Compared with the HS group, the HE group showed lower body weight and fasting blood glucose beginning at the second and sixth week, respectively. The fat weight, fat mass ratio and adipocyte areas of eWAT were significantly increased in the HS group compared to the NS group after 12 weeks of HFS diet. Compared with the HS group, the above alterations were rectified in the HE group (Figure 2C,D).

**Figure 3:**
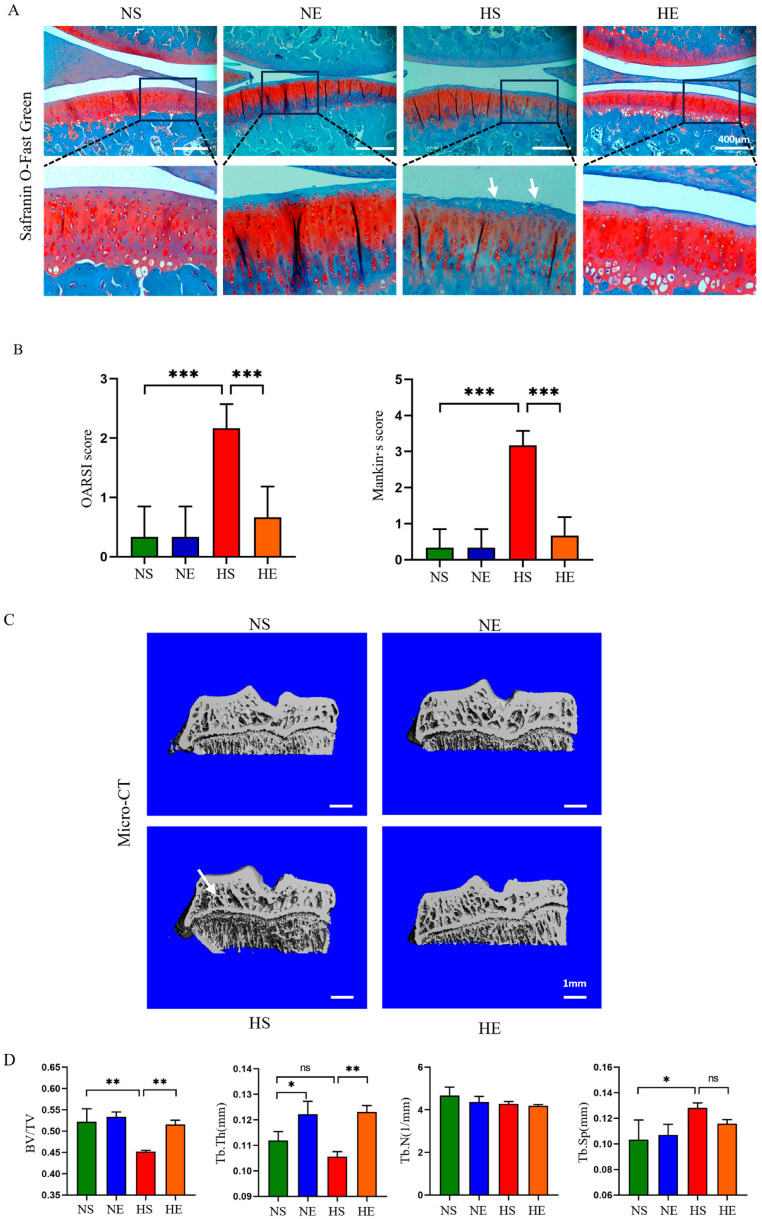
Exercise prevents joint degradation induced by HFS diet. (**A**,**B**) Safranin O-Fast Green of sagittal section (scale bar = 400 μm), OARSI and Mankin scores of each group. White arrows indicate superficial cartilage erosion and surface irregularities in the HS group, demonstrating articular cartilage degeneration. (**C**,**D**) Micro-CT images (scale bar = 1 mm) and parameters of the tibial subchondral bone of each group. White arrows indicate subchondral bone perforations and trabecular rarefaction in the HS group, demonstrating bone loss of subchondral bone. Data are presented as the mean ± SD, *n* = 6. ns: not significant, * *p* < 0.05, ** *p* < 0.01, *** *p* < 0.001.

Considering that obesity is a risk factor for MetS-OA, we further observed the alterations in articular cartilage and subchondral bone. As shown in Figure 3A, the HS group exhibited irregular articular surfaces with cleft formation, focal cartilage loss, uneven matrix staining, and diminished safranin-O intensity compared with the NS group, indicative of extracellular matrix degradation and chondrocyte deterioration. Quantitative histological scoring using OARSI and modified Mankin criteria confirmed substantially elevated degenerative indices in the HS group relative to NS controls (Figure 3B). Micro-CT results revealed subchondral bone lesions in the HS group. The parameters of subchondral trabecular bone, including bone volume fraction (BV/TV) and trabecular thickness (Tb.Th) were lower than the NS group (Figure 3C,D). These observations collectively underscore the structural degeneration of the cartilage–subchondral bone functional unit in the HFS-induced model. After 12 weeks of treadmill walking, the degeneration of cartilage was relieved in the HE group compared to the HS group (Figure 3A,B). Concomitantly, subchondral bone remodeling showed favorable modulation, evidenced by diminished bone resorption and recuperated BV/TV and Tb.Th parameters (Figure 3C,D). Quantitative analysis of quadricep muscle and gastrocnemius muscle fiber cross-sectional area (CSA) showed no significant difference among the four groups (Supplementary Figure S1). These findings demonstrate that exercise prevents the emergence of OA-like phenotypes induced by a HFS diet.

### 3.2. Exercise Prevents the Dysbiosis of Gut Microbiota Induced by HFS Diet

**Figure 4:**
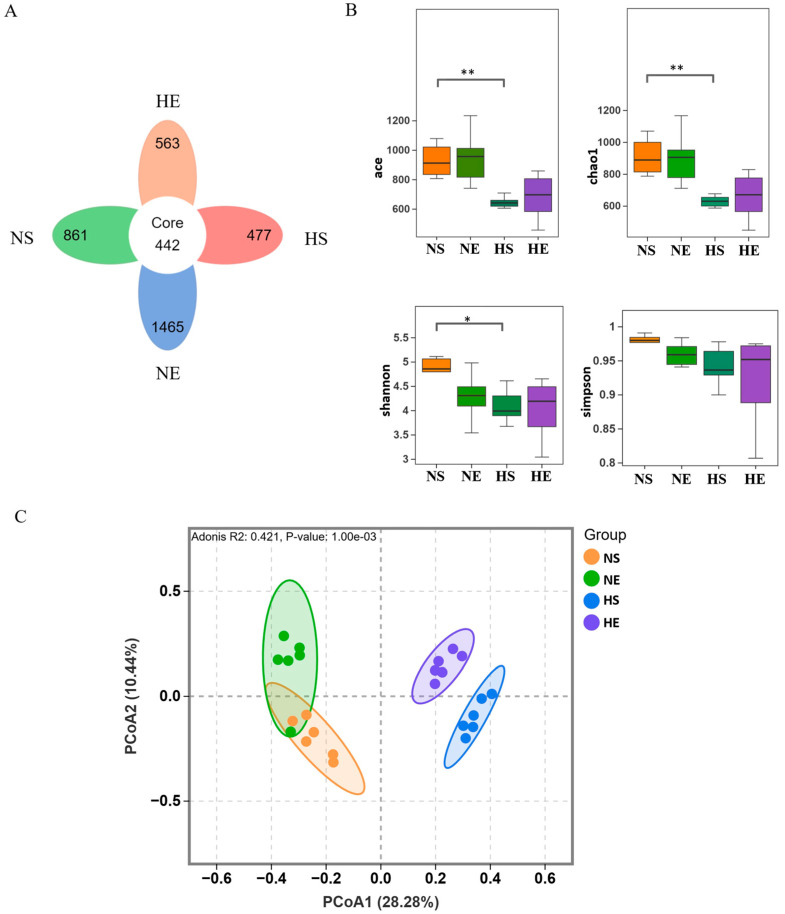
The diversity of gut microbiota in rats induced with the HFS diet. (**A**) The Venn diagram shows the common and unique ASVs of each group. (**B**) α diversity including Simpson, Shannon, ACE and Chao1 index. (**C**) PCoA analysis of gut microbiota based on Bray–Curtis distance. Each point represents an individual biological sample (*n* = 6). Data are presented as the mean ± SD. ns: not significant, * *p* < 0.05, ** *p* < 0.01.

**Figure 5:**
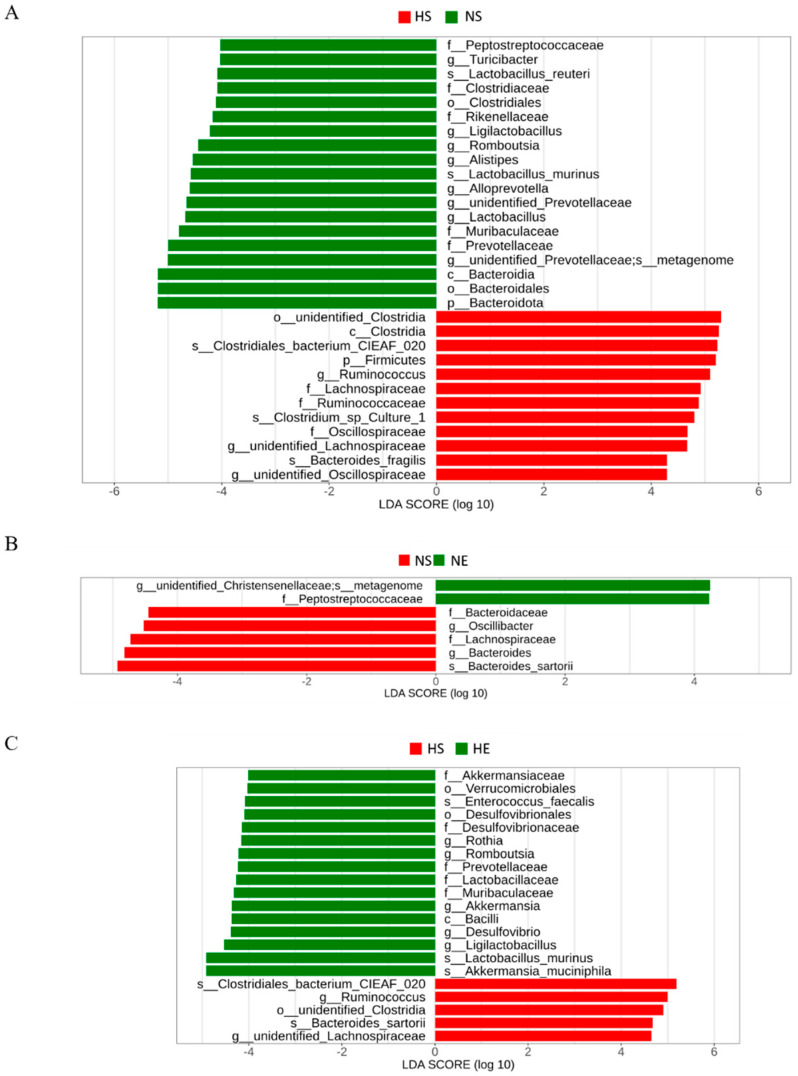
The LEfSe analysis among different groups. (**A**) NS vs. HS. (**B**) NS vs. NE. (**C**) HS vs. HE. *n* = 6. LDA ≥ 4, *p* < 0.05.

To further investigate the potential mechanism by which exercise protects against joint degeneration induced by the HFS diet, the gut microbiota was assessed by 16S rRNA sequencing. Following quality control, 24 fecal samples yielded a mean of 106,727 raw reads (range: 97,860–136,763) and 92,230 effective reads (range: 73,399–114,457) per sample, with a mean filtering rate of 13.6% and low variation in sample size (CV < 11%). Rarefaction curves for all samples reached a plateau, indicating sufficient sequencing coverage for downstream diversity analysis. As shown in Figure 4A, 442 ASVs existed in all groups, thus defined as core ASVs and 861, 1465, 477, and 563 ASVs were uniquely identified in the NS, NE, HS and HE groups (Figure 4A). α diversity analysis was performed on the taxonomically annotated ASV dataset. As shown in Figure 4B, compared with the NS group, the Shannon, ACE and Chao1 indices were decreased in the HS group. PCoA based on Bray–Curtis distance displayed the overall separation pattern of gut microbiota among groups (Figure 4C). PERMANOVA analysis further revealed significant differences between all group pairs (*p* < 0.05) except NS vs. NE (Supplementary Table S2). The LEfSe analysis (LDA score ≥ 4.0, *p* < 0.05) identified taxa with significantly differential relative abundance across groups. As shown in Figure 5, the HS group was significantly enriched in phylum *Firmicutes*, class *Clostridia*, unidentified order *Clostridia*, families *Lachnospiraceae*, *Ruminococcaceae* and *Oscillospiraceae*, genus *Ruminococcus*, unidentified genera *Lachnospiraceae* and *Oscillospiraceae*, and species *Clostridiales_bacterium_CIEAF_020*, *Clostridium_sp_Culture_1* and *Bacteroides_fragilis* compared with the NS group. The HE group was significantly enriched in class *Bacilli*, orders *Verrucomicrobiales* and *Desulfovibrionales*, families *Akkermansiaceae*, *Desulfovibrionaceae*, *Prevotellaceae*, *Lactobacillaceae* and *Muribaculaceae*, genera *Rothia*, *Romboutsia*, *Akkermansia*, *Desulfovibrio* and *Ligilactobacillus*, and species *Enterococcus_faecalis*, *Lactobacillus_murinus* and *Akkermansia_muciniphila* compared with the HS group.

**Figure 6:**
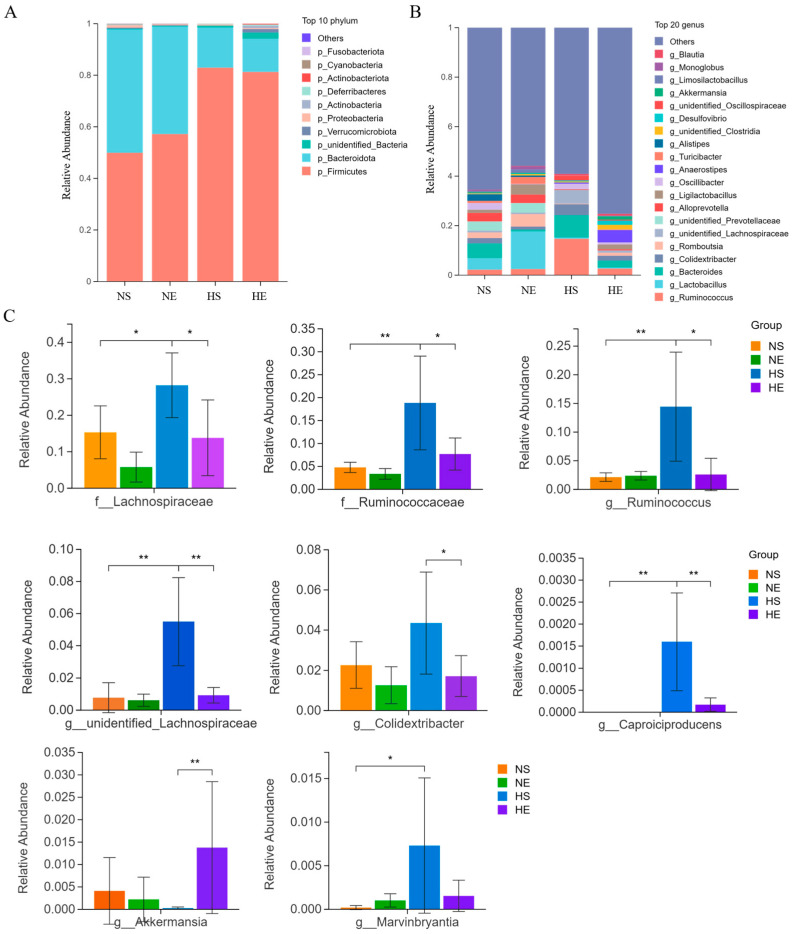
Exercise modulates the composition of gut microbiota induced by HFS diet. (**A**,**B**) The species stack bar chart shows the top 10 phyla and the top 20 genera. (**C**) Differences between groups at family and genus level. Data are presented as the mean ± SD, *n* = 6. * *p* < 0.05, ** *p* < 0.01.

In order to further investigate the effect of exercise on the composition of the gut microbiota, differences between groups at phylum, family and genus levels were further analyzed. According to the results, the top 10 phyla and top 20 genera with the highest abundance were selected for display (Figure 6A,B). At the phylum level, compared with the NS group, the relative abundance of phylum *Firmicutes* was significantly increased and phylum *Bacteroidota* was significantly decreased in the HS group as well as the increased *Firmicutes/Bacteroidota* ratio (Supplementary Figure S2). At the family level, the elevation of the HFS-diet-induced families *Lachnospiraceae* and *Ruminococcaceae* was prevented in the HE group as a result of treadmill walking. At the genus level, HS-group rats exhibited increased genera *Ruminococcus* and *Caproiciproducens* and unidentified genus *Lachnospiraceae* compared to the NS group. Interestingly, the increased gut microbiota mentioned above was rectified in the HE group. In addition, genus *Colidextribacter* was decreased and genus *Akkermansia* was increased after treadmill walking in the HE group compared to the HS group. Overall, these results indicate that treadmill walking can modify the β diversity and composition of gut microbiota induced by a HFS diet.

### 3.3. Exercise Prevents the Reduction in AA Induced by a HFS Diet

**Figure 7:**
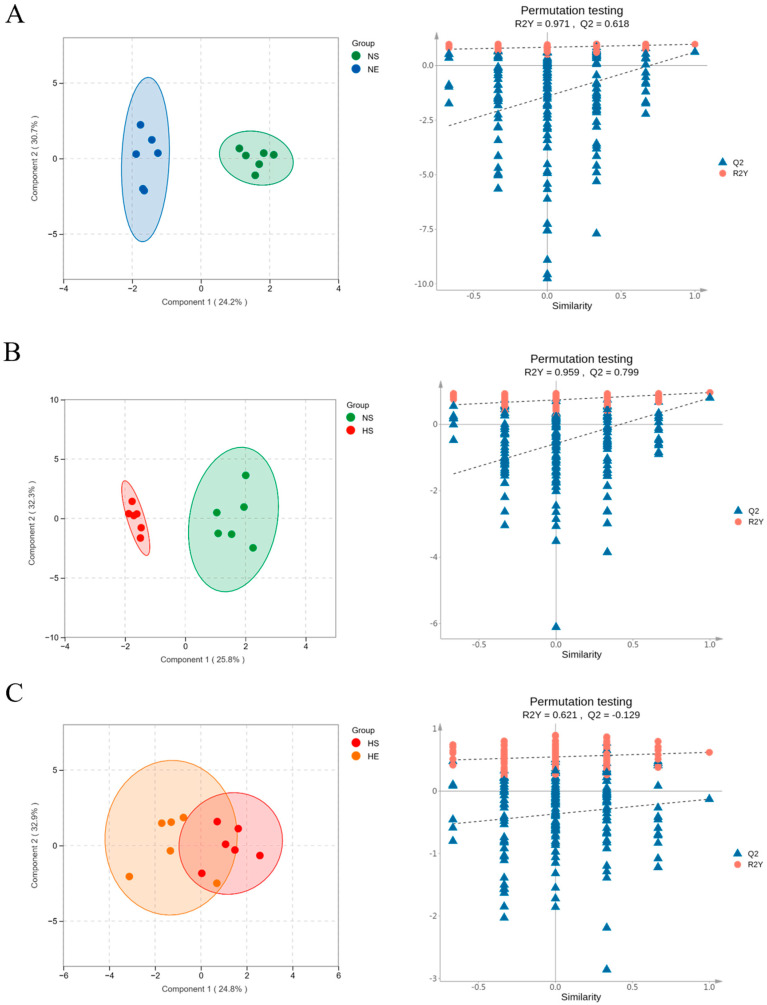
The OPLS-DA model of SCFAs between groups and the validation of the OPLS-DA model via permutation tests between groups. (**A**) NS versus NE. (**B**) NS versus HS. (**C**) HS versus HE. *n* = 6.

**Figure 8:**
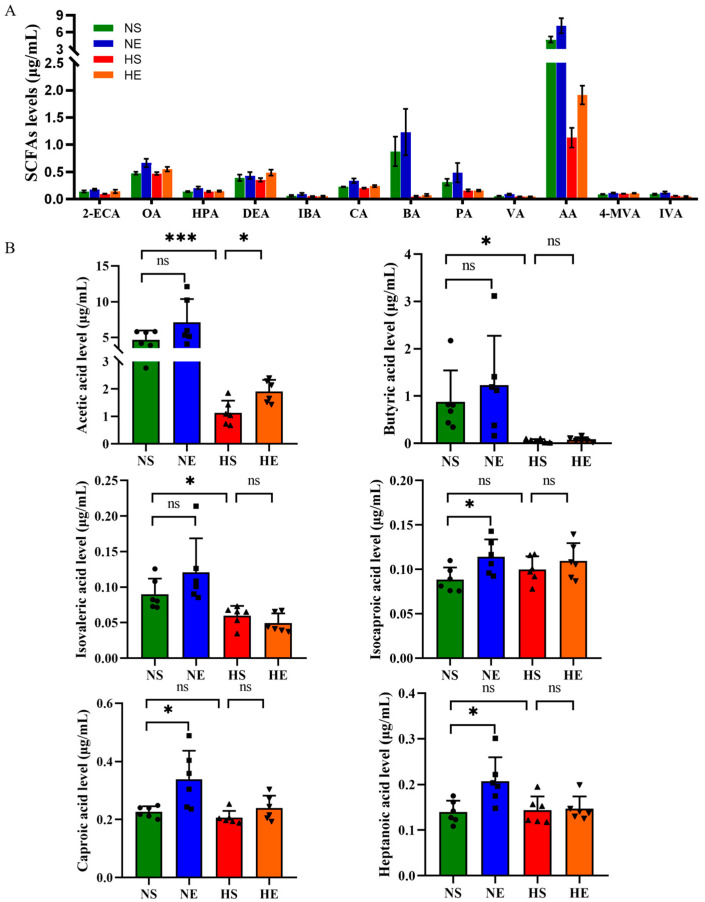
Exercise prevents the reduction in AA induced by the HFS diet. (**A**) The histogram shows the fatty chains detected in the four groups. (**B**) The histogram shows the content difference in each SCFA in different groups. (AA, acetic acid; BA, butyric acid; PA, propionic acid; IBA, isobutyric acid; VA, valeric acid; IVA, isovaleric acid; CA, caproic acid; HPA, heptanoic acid; 2-ECA, 2-ethylcaproic acid; OA, octanoic acid; DEA, decanoic acid; 4-MVA, isocaproic acid.) Data are presented as the mean ± SD, *n* = 6. ns: not significant, * *p* < 0.05, *** *p* < 0.001.

To investigate whether SCFAs mediate the beneficial effects of exercise, we quantified serum SCFA levels by GC-MS. OPLS-DA revealed clear separation between the NS and HS groups, as well as between the NS and NE groups, indicating robust model reliability (Figure 7A,B). In contrast, although the HS and HE groups exhibited a visual separation trend in the score plot, the OPLS-DA model failed to achieve predictive validity (Q^2^ = −0.129), suggesting that the overall SCFA metabolic profiles between these two groups were not robustly distinguishable (Figure 7C). As the global metabolic signature failed to robustly discriminate between the HS and HE groups, we subsequently performed univariate statistical analysis on the quantified data to pinpoint specific differentially abundant metabolites. Twelve fatty acids were detected across all samples (Figure 8, Supplementary Figure S3). Compared with the NS group, the HS group showed significantly reduced levels of acetic acid (AA), butyric acid (BA), and isovaleric acid (IVA). Notably, treadmill exercise attenuated the HFS-induced decrease in AA in the HE group (Figure 8B). Collectively, these results suggest that a HFS diet decreases specific SCFA levels, whereas exercise partially rescues AA reduction.

### 3.4. The Modification of AA Is Related to the Gut Microbiota and Joint Degeneration

**Figure 9:**
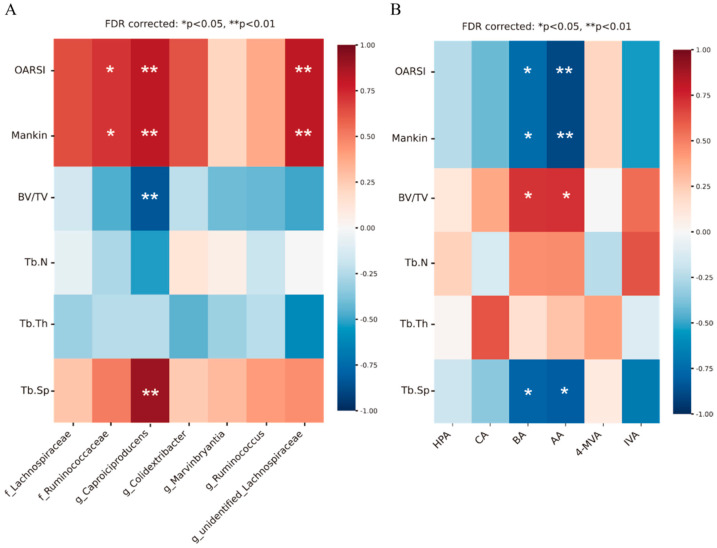
Modifications of gut microbiota and SCFAs are related to joint degeneration. (**A**) Heat map of spearman correlations between gut microbiota and joint structural parameters, including OARSI, Mankin score and micro-CT data. (**B**) Heat map of spearman correlations between SCFAs and joint structural parameters. *N* = 24. * *p* < 0.05, ** *p* < 0.01.

**Figure 10:**
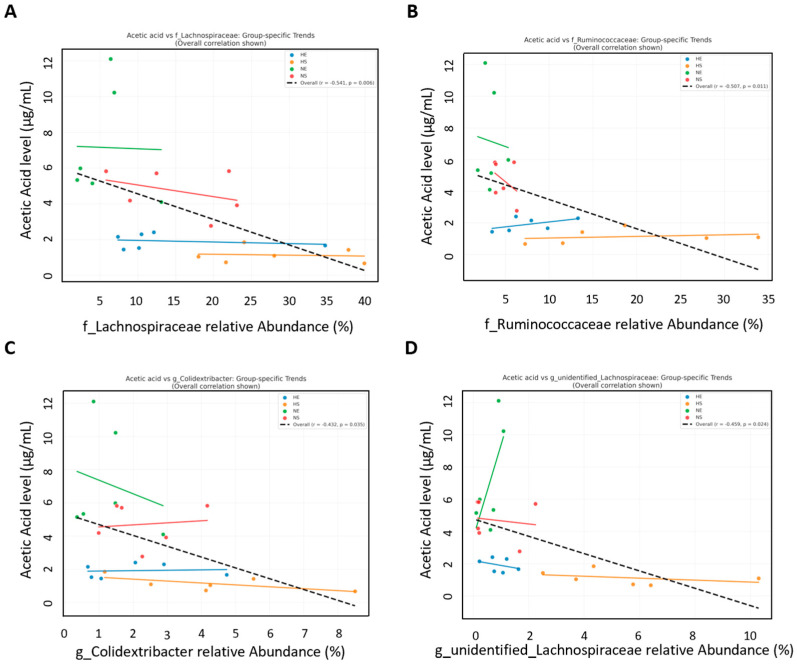
Scatter plot of the correlation between AA and gut microbiota. (**A**) AA vs. family *Lachnospiraceae*. (**B**) AA vs. family *Ruminococcaceae*. (**C**) AA vs. genus *Colidextribacter*. (**D**) AA vs. unidentified genus *Lachnospiraceae*. *N* = 24.

Due to the findings that exercise affected gut microbiota, SCFAs and joint degeneration separately, we further investigate the relationship between them through Spearman’s correlation analysis. As shown in Figure 9A, the family *Ruminococcaceae*, genus *Caproiciproducens* and unidentified genus *Lachnospiraceae* were positively correlated with OARSI and Mankin scores. Genus *Caproiciproducens* was negatively correlated with BV/TV and positively correlated with trabeculae separation (Tb.Sp). Moreover, correlations were identified between SCFAs, joint structural features, and gut microbiota. As shown in Figure 9B, AA and BA were significantly negatively correlated with OARSI, Mankin score and Tb.Sp and positively correlated with BV/TV. In addition, AA was significantly negatively correlated with families *Lachnospiraceae* and *Ruminococcaceae*, genus *Colidextribacter* and unidentified genus *Lachnospiraceae* in the overall correlation analysis (Figure 10). These findings indicate that exercise-induced alterations in gut microbiota and elevation in AA were strongly associated with the observed joint protection.

## 4. Discussion

OA is a complex, multifactorial degenerative disease involving various components of the joint [46]. Obesity is one of the important risk factors for OA [47]. Diet-induced obesity typically develops within 10–12 weeks, leading animals to reach a state of “allostasis” characterized by increased body weight and adiposity [48]. Obesity-related bioactive factors, particularly altered adipokines, can promote the development and progression of OA by enhancing the expression of inflammatory cytokines and degradative enzymes, suppressing cartilage matrix synthesis, and affecting subchondral bone remodeling [49]. The gut microbiome is widely recognized as a source of diverse molecules, including enzymes and pro-inflammatory metabolites, which translocate from the compromised intestinal barrier into systemic circulation, thereby triggering systemic inflammation [14,50]. In this context, the hypothesis that external interventions targeting gut microbiota and their derived metabolites could potentially mitigate OA progression is plausible.

Exercise has been demonstrated to be a highly effective intervention for OA, particularly in improving joint function and alleviating pain. Our previous study demonstrated that exercise can modulate the composition of disease-related gut microbiota, increase beneficial metabolites, and ameliorate articular cartilage degeneration [16]. The aim of this study was to identify potential associations among gut microbiota, specific gut microbiota-derived metabolites (SCFAs), and OA severity in the context of obesity-associated OA. Our findings suggest that exercise contributes to maintaining the integrity of joint cartilage and subchondral bone, effectively slowing down the development of OA induced by a HFS diet. The potential connection between exercise and OA development may be associated with modulating disruptions in the gut microbiota and increased AA levels among SCFAs.

First, using 16S rRNA gene sequencing, we investigated the impact of exercise and a HFS diet on the structure and composition of the gut microbiota. The interaction between diet and the gut microbiota promotes the development and progression of metabolic syndrome through multiple mechanisms, including enhanced energy harvest from the diet, altered energy homeostasis, increased production of SCFAs, gut microbiota-induced metabolic endotoxemia, systemic low-grade inflammation, oxidative stress, and modulation of signaling pathways and the immune system [51]. Gut microbial metabolites, such as SCFAs, bile acids, and tryptophan, exert regulatory effects on intestinal barrier integrity, immune balance, and bone destruction [52]. LPS can translocate into systemic circulation and exert detrimental effects on organs and joints through the induction of low-grade systemic inflammation [53]. The restoration of the gut microbiota is crucial for improving chronic inflammation and metabolic disturbances [54,55]. Numerous studies have shown changes in the gut microbiota of obesity mice models, especially an increase in phylum *Firmicutes* and a decrease in phylum *Bacteroidetes*, which aligns with our study results [35,56,57]. Compared to the NS group, our study found an upregulation of phylum *Firmicutes* and a downregulation of phylum *Bacteroidota* in the HS group. In addition, compared with the NS group, HS-group rats displayed a significant increase in the abundance of families *Lachnospiraceae* and *Ruminococcaceae*, while exercise reversed the aberrant gut microbiota composition. Furthermore, the abundance of family *Ruminococcaceae* was positively correlated with OARSI and Mankin scores, indicating a potential link between this bacterial family and OA severity. This finding is consistent with previous work by Huang et al., who reported that family *Ruminococcaceae* abundance was associated with OA severity and elevated levels of inflammatory factors such as IL-1β and IL-6 [58]. Thus, the decreased family *Ruminococcaceae* may interpret the positive anti-inflammatory effect of exercise in OA. Similarity, we observed an increase in unidentified genus *Lachnospiraceae* in the HS group, aligning with reports of its elevation in high-fat-diet-fed mice [59,60]. Exercise intervention in the HE group suppressed the increase in unidentified genus *Lachnospiraceae*. Meanwhile, the abundance of unidentified genus *Lachnospiraceae* was positively correlated with OARSI and Mankin scores. These results are consistent with the hypothesis that the protective effect of exercise on OA may be associated with the gut microbiota in rats, although a causal mechanism remains to be established.

Next, we measured the levels of SCFAs using GC-MS analysis. SCFAs are key microbial metabolites produced through anaerobic fermentation and serve as important metabolites involved in various biological functions, including host metabolism, gut function, and immunity [22,23,61]. SCFAs exert chondroprotective effects by targeting NF-κB, MAPK, and PI3K signaling cascades, thereby attenuating inflammatory and oxidative insults to chondrocytes while preserving cartilage matrix integrity [62]. In the present study, a significant downregulation of AA and BA was observed in rats following 12 weeks of a HFS diet. However, AA levels were restored after exercise intervention. Furthermore, inverse correlations were observed between AA levels and OA-related structural indices (Tb.Sp, OARSI, and Mankin scores), while a positive correlation was detected with BV/TV. AA represents the most predominant SCFA, comprising 60–75% of the total SCFA pool in circulation [63]. Deng et al. found AA displayed a robust positive association with bone mineral density (BMD) and BV/TV of subchondral bone in a mice OA model [64]. AA supplementation improved cartilage damage, and reduced synovial hyperplasia and inflammatory cell infiltration [65]. These results suggest that the joint protection observed in exercised rats is associated with restored AA levels. Correlation analysis further identified that four specific bacterial taxa (families *Lachnospiraceae*, *Ruminococcaceae*, genus *Colidextribacter* and unidentified genus *Lachnospiraceae*) had an overall negative correlation with AA. Consistent with our finding, Hu et al. reported that the abundance of families *Lachnospiraceae* and *Ruminococcaceae* within phylum *Firmicutes* increases, while the level of AA decreases in mice fed a high-salt diet [66]. Ma et al. documented a negative correlation between genus *Colidextribacter* and AA [67]. Gu et al. also found that unidentified genus *Lachnospiraceae* is associated with various intestinal metabolites [59]. These findings provide evidence that the modification in AA is strongly associated with gut microbiota and joint degeneration.

Although our research has demonstrated that exercise can regulate the composition of the gut microbiota and AA, preventing HFS-diet-induced obesity-associated OA, there are still some limitations in our study. The 16S rRNA sequencing technology utilized in our study is largely confined to bacterial classification and does not precisely identify specific microbial species and strains. To address this limitation, employing shotgun metagenomic sequencing could provide robust estimates of microbial communities and detailed functional annotations for further investigation. Additionally, the proposed mechanism should be regarded as a speculative hypothesis that requires direct experimental validation. Employing multi-omics data analysis and more sophisticated techniques such as fecal microbiota transplantation (FMT) can elucidate the relationship between gut microbiota and AA in the progression of OA. SCFA quantification was limited to serum, which reflects systemic availability but does not directly capture local intestinal microbial fermentative activity; future studies should incorporate simultaneous fecal and serum SCFA profiling to better assess gut microbiota metabolic output. Furthermore, the interpretation of the study results and the description of identified bacteria and AA as biomarkers for OA in rats are challenging due to limited sample sizes and potential fluctuations in the gut microbiome. Moreover, this relationship may be influenced by unmeasured confounders (e.g., dietary intake, body weight fluctuations, and stress levels) and is further limited by the single-sex, single-species design. Therefore, our study results can only provide preliminary insights into its mechanisms and require validation in large-scale longitudinal studies.

## 5. Conclusions

In summary, our findings suggest that exercise is associated with attenuated OA progression, altered gut microbiota composition, and restoration of AA levels in the HFS-induced rats. Moreover, these observations raise the possibility that the beneficial effects of exercise on joint integrity may be linked to gut microbial shifts and increased AA availability. These findings provide novel theoretical insights into the mechanisms underlying exercise-mediated joint improvement.

It should be noted, however, that the preclinical model employed in this study served as a preliminary investigation into the correlations among gut microbiota, AA, and OA progression. Therefore, rigorously controlled clinical trials and large-scale prospective studies are warranted to establish causal relationships among gut microbiota, AA, and OA progression. Ultimately, this exploratory framework highlights a promising direction for developing therapeutic strategies targeting AA or the gut microbiota in OA management.

## Data Availability

The datasets used and/or analyzed during the current study are available from the corresponding author Xu Tao on reasonable request. The raw data were deposited in NCBI Sequence Read Ar-chive (SRA) (accession numbers for NCBI: BioProject: PRJNA1231086 for 16S rRNA sequencing).

## Supplementary Materials

The following supporting information can be downloaded at https://www.mdpi.com/article/10.3390/metabo16070452/s1, Table S1: Primers for 16S rRNA sequencing; Table S2: The ADONIS analysis based on Bray–Curtis distance; Figure S1: HE staining of muscles in each group; Figure S2: Differences between groups at phylum level; Figure S3: Histogram shows the content difference in each SCFA in different groups.
