# Supplementary material for: Gut–Joint Axis: The Role of Exercise on Gut Microbiota and Acetic Acid Modulation in Obesity-Associated Osteoarthritis Rats
Source: Metabolites. 2026 Jun 27;16(7):452. doi: 10.3390/metabo16070452 (PMC13413777; doi:10.3390/metabo16070452)
Supplement: Supplementary file 1 [file metabolites-16-00452-s001.zip › metabolites-4281428-supplementary.pdf]

Supplemental table S1. Primers for 16S rRNA sequencing

Primer Name	Primer Sequence (5'-3')
341F	CCTAYGGGRBGCASCAG
806R	GGACTACNNGGGTATCTAAT

Supplemental table S2. The ADONIS analysis based on Bray-Curtis distance.

Group	Df	Sums OfSqs	Mean Sqs	F.Model	R2	Pr (>F)	Bonferroni-corrected p
NS vs NE	1(10)	0.538 (1.976)	0.538 (0.1976)	2.72	0.214 (0.786)	0.015	0.090
NS vs HS	1(10)	1.262 (1.979)	1.262 (0.1979)	6.38	0.389 (0.611)	0.007	0.042
NS vs HE	1(10)	1.096 (2.262)	1.096 (0.2262)	4.846	0.326 (0.674)	0.002	0.012
NE vs HS	1(10)	1.585 (1.955)	1.585 (0.1955)	8.105	0.448 (0.552)	0.003	0.018
NE vs HE	1(10)	1.192 (2.239)	1.192 (0.2239)	5.326	0.348 (0.652)	0.001	0.006
HS vs HE	1(10)	0.555 (2.241)	0.555 (0.2241)	2.476	0.198 (0.802)	0.005	0.030

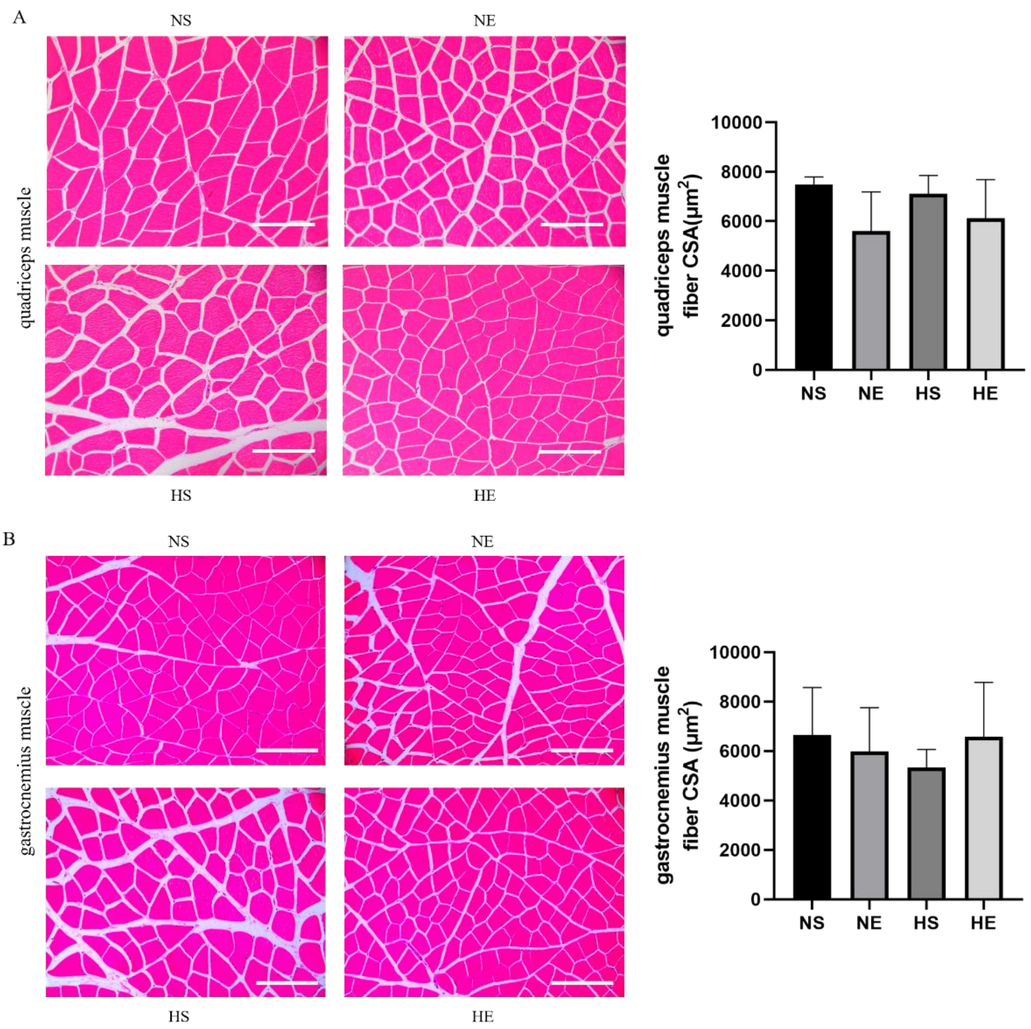

Supplemental figure S1. HE staining of muscles in each group. A: quadriceps muscle; B: gastrocnemius muscle (scale bar = 200 μm).

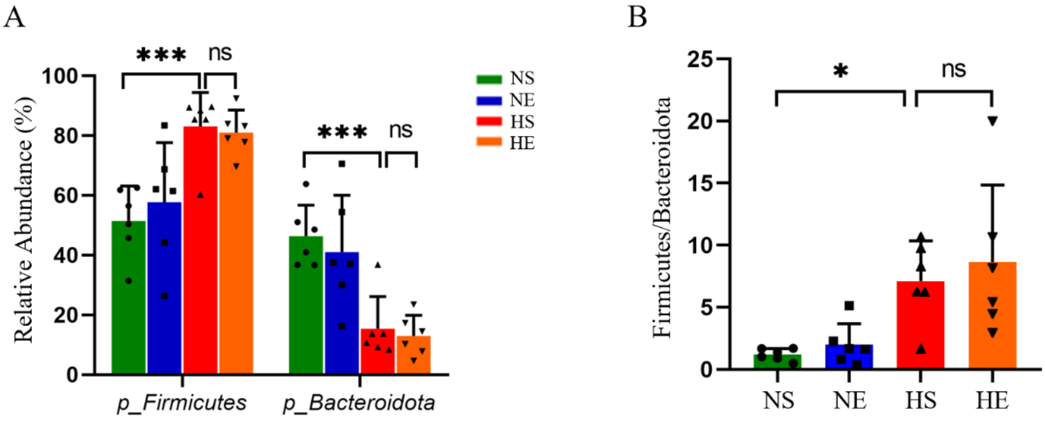

Supplemental figure S2. Differences between groups at phylum level. A: p_*Firmicutes* and p_*Bacteroidota*; B: *Firmicutes/Bacteroidota*. Data are presented as the mean ± SD. ns: not significant, *$p < 0.05$, *** $p < 0.001$.

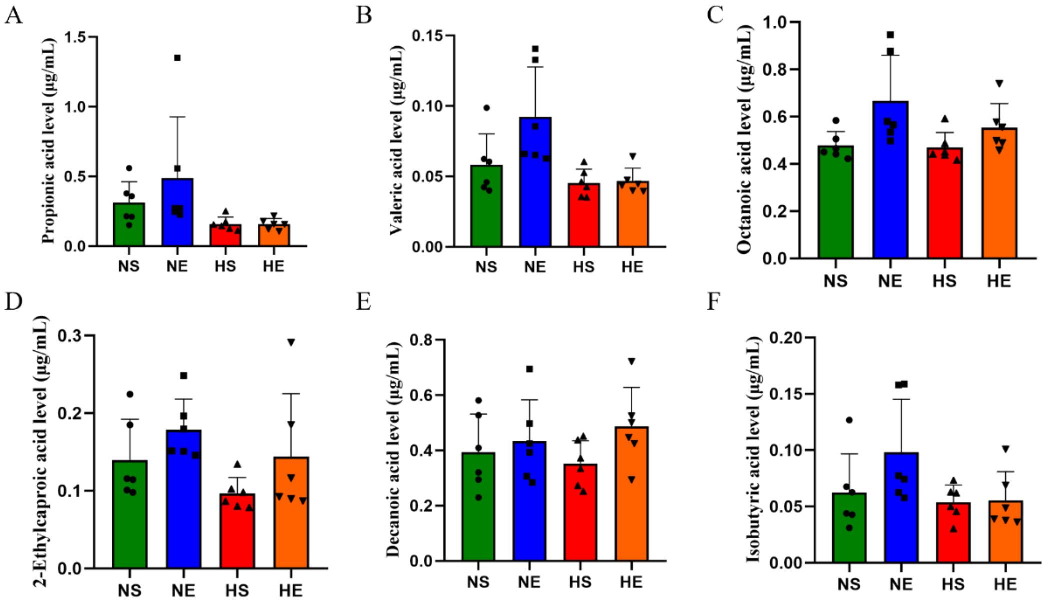

Supplemental figure S3. Histogram shows the content difference of each SCFAs in different groups. A: PA; B: VA; C: OA; D:2-ECA; E: DEA; F: IBA. n=6.
